# Genetic structure of *Aedes albopictus* (Diptera: Culicidae) populations in China and relationship with the knockdown resistance mutations

**DOI:** 10.1186/s40249-023-01096-x

**Published:** 2023-05-05

**Authors:** Wenqi Shan, Hao Yuan, Hanming Chen, Haowei Dong, Qiuming Zhou, Feng Tao, Jie Bai, Huiying Chen, Yajun Ma, Heng Peng

**Affiliations:** 1 Department of Naval Medicine, Naval Medical University, Shanghai, 200433 China; 2 Department of Medical Microbiology and Parasitology, College of Basic Medical Sciences, Naval Medical University, Shanghai, 200433 China

**Keywords:** *Aedes albopictus*, Population structure, Microsatellite, Knockdown resistance, China

## Abstract

**Background:**

Mosquito control is needed to prevent dengue fever, which is mainly spread by *Aedes albopictus* in China. Application of insecticides is one of the main mosquito control methods; however, this approach can fail due to the knockdown resistance (*kdr*) gene mutation that causes decreased sensitivity to insecticides in *Ae. albopictus*. The *kdr* mutation patterns among different regions in China differ significantly. However, the underlying mechanism and factors that influence *kdr* mutation remain unclear. To explore the potential influence of genetic background on the development of insecticide resistance in *Ae. albopictus*, we analyzed the genetic structure of *Ae. albopictus* populations in China and its correlation with major *kdr* mutations*.*

**Methods:**

We collected *Ae. albopictus* from 17 sites in 11 provinces (municipalities) across China from 2016 to 2021 and extracted the genomic DNA from individual adult mosquitoes. We selected eight microsatellite loci for genotyping, and based on microsatellite scores, we estimated intraspecific genetic diversity, population structure, and effective population size. The association between the intrapopulation genetic variation and F1534 mutation rate was evaluated by the Pearson correlation coefficient.

**Results:**

Based on variation analysis of the microsatellite loci of 453 mosquitoes representing 17 populations throughout China, more than 90% of the variation occurred within individuals, whereas only about 9% of the variation occurred among populations, indicating that field populations of *Ae. albopictus* are highly polymorphic. The northern populations tended to belong to gene pool I (BJFT 60.4%, SXXA 58.4%, SDJN 56.1%, SXYC 46.8%), the eastern populations tended to belong to pool III (SH 49.5%, JZHZ 48.1%), and the southern populations tended to belong to three different gene pools. Moreover, we observed that the greater the fixation index (*F*_ST_), the lower the wild-type frequency of F1534 of *VSGC*.

**Conclusions:**

The degree of genetic differentiation among *Ae. albopictus* populations in China was low. These populations were divided into three gene pools, in which the northern and eastern pools are relatively homogeneous, while the southern gene pool is heterogeneous. The possible correlation between its genetic variations and *kdr* mutations is also noteworthy.

**Graphical Abstract:**

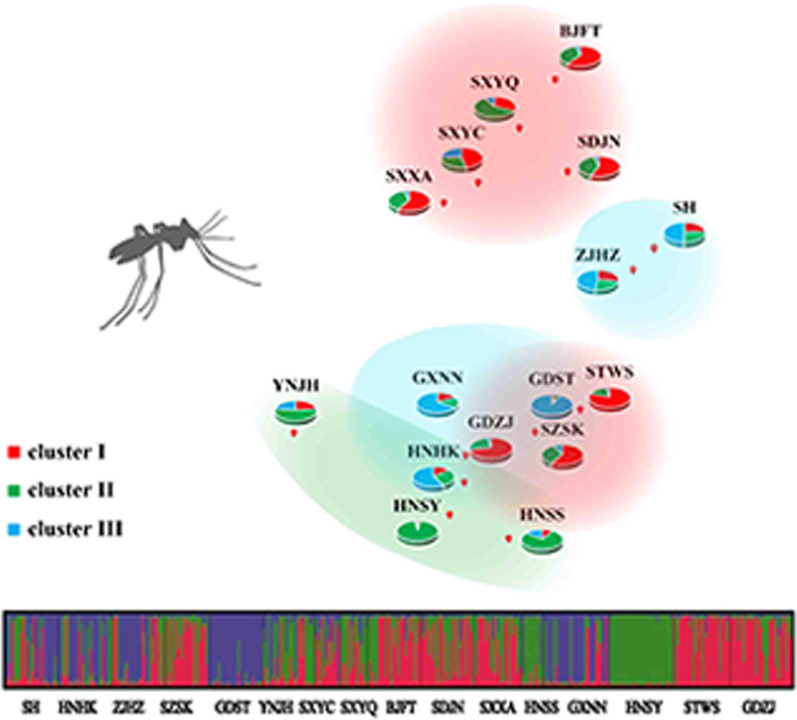

**Supplementary Information:**

The online version contains supplementary material available at 10.1186/s40249-023-01096-x.

## Background

Dengue fever has become a public health emergency in China. In 2019, over 22,000 dengue cases were reported, and since then, the distribution of the disease has been significantly expanding [1, 2]. *Aedes albopictus* is widely distributed in China, where it can be found from Liaoning Province in the north to Hainan Province in the south, an area that includes tropical, subtropical, and temperate climate zones [3]. *Ae. albopictus* is the main vector of dengue fever in China due to its abundance and wide distribution [4].

Mosquito control is an important component of the integrated programs designed to prevent dengue fever transmission, and insecticide spraying is one of the most commonly used methods of mosquito control. This method is simple and quickly effective, thus it has been a critical component of emergency control of mosquito-borne diseases, including dengue fever. Because of its importance in the prevention and control of mosquito-borne infectious diseases, the development of insecticide resistance in mosquitoes has become a focal issue. The mechanisms of insecticide resistance in mosquitoes include behavioral resistance, target insensitivity, and metabolic detoxification, with target insensitivity being considered the main mechanism of insecticide resistance in *Ae. albopictus*. The well-known knockdown resistance (*kdr*) is caused by mutations in the voltage-gated sodium cannel (*VGSC*) gene, which encodes the target site of pyrethroids [5]. The types of *kdr* mutations found in field populations of *Ae. albopictus* include F1534S, F1534C, I1532T, and V1016G. In China, the mutation rate of *kdr* in field populations of *Ae. albopictus* is extremely high, indicating that it undergoes rapid evolution.

The origin of insecticide resistance in mosquitoes has long been a topic of interest. There is evidence indicating that mosquito population structure and insecticide resistance are two mutually influencing factors contributing to insecticide resistance. Chang et al. demonstrated the impact of insecticide selection factors on the population structure of *Anopheles sinensis* in China [6]. Rahman et al. also observed prevalent *kdr* mutations, low genetic architecture, and frequent migration in *Ae. aegypti* populations in Rio de Janeiro, Brazil [7]. In our previous study, we found that the *kdr* mutation pattern of the *Ae. albopictus* population in China is correlated with the average annual temperature at the collection site [8]. The research showed that *Ae. albopictus* populations in tropical, subtropical, and temperate regions of China have different genetic structure characteristics [9]. These studies have given rise to our interest in exploring the association between population genetic structure and insecticide resistance in *Ae. albopictus*.

Previous research on the molecular population genetic structure of *Ae. albopictus* has mainly focused on its invasion and migration routes [10–13]. These studies have applied the following molecular markers: allozyme, random amplified polymorphic DNA [14], NADH dehydrogenase subunit 5 [15–17], cytochrome c oxidase subunit I (COI) [18–22], cytochrome B [23] of mitochondrial DNA, ribosomal DNA ITS2 [24], and microsatellite DNA [9, 10, 25–28]. Analysis of COI sequences and nuclear microsatellites in *Ae. albopictus* populations found substantial intermixing between Papua New Guinea’s southern Fly region and Torres Strait Island, which has been attributed to human sea traffic shuttling this mosquito between the two sites; and this extensive movement may well compromise *Ae. albopictus* eradication attempts in this region [11]. In Vietnam, the COI haplotype grouping of *Ae. albopictus* found geographically distributed populations, resulting in a distinct population structure in which northeastern populations and the remaining populations were genetically differentiated [20]. Microsatellite DNA analysis of *Ae. albopictus* populations in East Asia, the Indian Ocean, the Mediterranean Basin, the Pacific Ocean, and North America suggests that independent and transcontinental introductions may have facilitated the rapid establishment of adventive populations through the admixture of unrelated genomes. This study found a high level of intrapopulation variability, and this variability may extend to the genetic mechanisms controlling vector competence [10].

The population genetic structure of *Ae. albopictus* in some areas of China has been studied using molecular markers [9, 10, 29], and COI analysis indicates that gene flow occurs commonly among 15 populations in Guangzhou, Guangdong; moreover, the analysis found no genetic differentiation nor differences in genetic diversities among populations [30, 31]. In contrast, there was an obvious genetic differentiation between *Ae. albopictus* in Hainan and a few samples collected from 10 provinces in China [32]. These studies indicate the need to more fully study the genetic structure of *Ae. albopictus* populations in China, and the relationship between genetic structure and insecticide resistance development also needs to be investigated.

In this study, we analyzed the genetic structure of *Ae. albopictus* in China by collecting samples from 17 field populations located in regions ranging from temperate to tropical. We analyzed the population genetic structure of *Ae. albopictus* based on based on 8 polymorphic microsatellite loci. We then analyzed the correlation between genetic variations within *Ae. albopictus* populations and *kdr* mutations. The main objectives of this study were to estimate the degree of genetic differences among *Ae. albopictus* populations in China; and to analyze the factors influencing insecticide resistance for the development of vector control strategies.

## Methods

### Sample collection and species identification

To encompass the distribution range of *Ae. albopictus* in China, we selected 17 sample collection sites within 11 provinces (municipalities) situated from the north to south of China. These sites include temperate, subtropical, and tropical climate regions. Samples were collected from 2016 to 2021 (Additional file 1: Table S1) by scooping larvae and pupae in breeding sites (more than 8 breeding sites in a collection site), and then bringing these back to the laboratory for rearing to adults under standard conditions [26 ± 1 °C, 65 ± 5% relative humidity, photoperiod of 12/12 h (light/dark)]. Adult mosquitoes in outdoor environments were collected using aspirators, BGtraps (Biogents AG, Regensburg, Germany), or light traps (Houji Dianzi, Dongguan, China). Details on sample collection are summarized in Additional file 1: Table S1 and Figure S2. Species of adult *Aedes* mosquitoes were identified based on morphological characteristics [3] and confirmed by analysis of molecular markers [33].

### Microsatellite genotyping

Genomic DNA was extracted from individual adult mosquitoes using the DNAzol reagent (Invitrogen, Thermo Fisher Scientific, Waltham, MA, USA) according to the manufacturer’s instructions. Nineteen microsatellite loci were initially selected from the literature [11, 34], and eight microsatellite loci were finally selected based on results of preliminary experiments on amplification efficiency and site polymorphism, and they consisted of Alb-di-4 (GenBank accession number: KF146971), Alb-di-6 (KF146972), Alb-tri-6 (KF146974), Alb-tri-41 (KF146983), Alb-tri-46 (KF146975), AealbA9 (DQ366022), AealbB52 (DQ366024), and AealbF3 (DQ366027) (Additional file 1: Table S2). Microsatellite loci were amplified according to detailed protocols described by Schuelke [34], with each PCR reaction containing three primers: a sequence-specific forward primer with an M13(-21) adapter (5′- TGT AAA ACG ACG GCC AGT -3′) conjugated to its 5′ end, a sequence-specific reverse primer, and a universal FAM-labeled M13(-21) primer. Amplified fragments were separated by capillary electrophoresis in an automatic sequencer (ABI 3770, Applied Biosystems, Foster City, CA) and fragment sizes were scored using GENOTYPER 3.7 software (Applied Biosystems, Foster City, CA).

### Analysis of genetic differences within a population

Genetic variation within the population in each locality was estimated in terms of average numbers of alleles (n_a_), the number of private alleles (n_p_), and frequency of private alleles (*A*_p_), which were obtained using GenAlEx 6.5 [35]. The average number of alleles and private alleles were also calculated at the individual level from the expressions n_a_/n and n_p_/n, respectively. The inbreeding index (*F*_IS_) was obtained using FSTAT V.2.9.3.2 [36]. Observed and expected heterozygosity and pairwise-*F*_ST_ [37] values were computed using Microsatellite Analyser V.4.05 [38]. The statistical significance of each *F*_ST_ value was assessed by comparing the observed value to the values obtained from 10,000 matrix permutations, followed by Bonferroni corrections. Linkage disequilibrium between pairs of loci in each sample (number of dememorizations 1000, 100 batches, 1000 iterations per batch) and deviations from Hardy–Weinberg equilibrium (HWE) at each locus/sample combination were examined with GENEPOP V. 4.2 [39], and the statistical significance was assessed following Bonferroni corrections [40]. The allelic polymorphic information content and null allele frequencies (*A*_n_) for each locus were estimated using CERVUS [41]. The significance of the regression examining the relationship between genetic differences and the geographic distance between sample pairs was tested using a Mantel test [42] with 100,000 permutations in GENEPOP V. 4.2 [39].

### Population genetic structure and demographic inference

The relationships among populations were assessed using Principal Coordinate Analysis in GenAlEx 6.5 [35]. Bayesian clustering analysis in STRUCTURE V 2.3.2 [43–45] was used to infer population genetic structure using the admixture model and assuming independent allele frequencies. The burn-in was set to 1,000,000 steps, followed by 1,000,000 Markov Chain Monte Carlo replications. All runs were repeated 20 times for each number of possible clusters (*K*), set between 1 and 16 (i.e., the number of samples). The appropriate number of genetic clusters was determined by plotting the log probability [L(K)] and Δ*K* across multiple runs [46] as implemented in STRUCTURE HARVESTER [47]. Finally, the programs CLUMPP [48] and DISTRUCT [49] were used to average replicate runs and to generate bar graphs of structure results, respectively. An analysis of molecular variance (AMOVA) was used to examine the distribution of genetic variation using GenAlEx 6.5. The longterm effective population size (*Ne*) was estimated using NEESTIMATOR 2.1 [50] based on the heterozygote excess and linkage disequilibrium methods.

### Correlation between genetic variation within a population and *kdr* mutation

The *F*_ST_ value, representing the intrapopulation genetic variation, was estimated by GENEPOP V. 4.2[39]. Mutant F1534S is the most common type of *kdr* mutation in *Ae. albopictus* in China, and it is significantly associated with the pyrethroid resistance phenotype [51]. The frequency of F1534S was obtained from our previous publication [8], which studied the same populations as those in this study. The correlations between the frequency of F1534 wild-type and* F*_ST_ within 14 *Ae. albopictus* populations were evaluated by Pearson correlation analysis. The linear regression was implemented using the “ggpmisc” and “ggplot2” package of R 4.2.2 (R Foundation for Statistical Computing, Vienna, Austria. URL https://www.R-project.org).

## Results

### Intraspecific variation

The scores obtained from eight microsatellite loci in 453 mosquitoes collected from 17 localities across China, displayed different levels of polymorphism in terms of the number of alleles, which ranged from 7 (AealbB52) to 27 (AealbA9) alleles. The number of alleles per locus in each population is provided in Additional file 1: Table S3. Analysis of these loci yields a mean polymorphic information content of 0.57, suggesting that they are sufficiently informative for assessing the degree of population variability and structuring of the populations [52]. The results of Fisher's exact tests after sequential Bonferroni corrections reveal significant departures from HWE in 14 out of 120 population/locus combinations. However, these departures were not concentrated at any one locus or in any population. We observed no consistent pattern of linkage disequilibrium between any particular pair of loci; therefore, the 8 selected loci are independent and their variability likely reflects genome-wide patterns across populations.

These eight loci were considerably variable across the 17 populations (Table 1), and the GDST population displayed the highest level of variation with an *H*_O_ of 0.57. The maximum mean number of alleles/individual was observed in the GDGZ population (n_a_/n = 0.58). Private alleles were detected in all populations, except for the SH, JZHZ, GDST, and SXYC populations, with the highest proportion of private alleles found in HNSS (n_p_/n = 0.31). Analysis of the variability distribution in and among the populations (AMOVA) showed that most variation (91%) occurred within individuals, whereas only about 9% of the total variation was detected among populations, indicating that the field populations of *Ae. albopictus* are highly polymorphic.

**Table 1:** Estimated genetic variability among 17 *Aedes albopictus* populations collected from different regions in China

Population	*n*	n_a_	n_a_/n	n_p_	n_p_/n	*A*p	*H*_O_	*H*_E_	*A*_n_
SH	30	6.38	0.21	0	0.00	0.00	0.32	0.65	0.48
HNHK	30	6.50	0.22	3	0.01	0.02	0.33	0.66	0.40
ZJHZ	30	6.38	0.21	0	0.00	0.00	0.35	0.64	0.37
SZSK	25	5.88	0.24	1	0.04	0.03	0.33	0.59	0.33
GDST	30	4.88	0.16	0	0.00	0.00	0.57	0.63	0.07
YNJH	29	7.25	0.25	4	0.14	0.02	0.36	0.66	0.33
SXYC	15	5.25	0.35	2	0.13	0.04	0.37	0.67	0.30
SXYQ	15	4.63	0.31	0	0.00	0.00	0.34	0.57	0.24
BJFT	30	5.50	0.18	1	0.03	0.02	0.41	0.62	0.00
SDJN	30	6.25	0.21	2	0.07	0.02	0.36	0.57	0.24
SXXA	26	6.50	0.25	2	0.08	0.03	0.36	0.56	0.24
GDGZ	10	5.75	0.58	1	0.10	0.05	0.44	0.65	0.20
HNSS	13	5.13	0.39	4	0.31	0.11	0.43	0.62	0.19
GXNN	37	6.25	0.17	2	0.05	0.03	0.42	0.67	0.27
HNSY	36	5.75	0.16	3	0.08	0.06	0.39	0.57	0.25
STWS	32	5.50	0.17	2	0.06	0.02	0.32	0.53	0.24
GDZJ	35	7.38	0.21	4	0.11	0.02	0.38	0.59	0.25

*n*_*a*_ mean number of alleles, *n*_*a*_*/n* mean number of alleles/individual, *n*_*p*_ number of private alleles, *n*_*p*_*/n* mean number of private alleles/individual, *Ap* mean frequency of private alleles, *H*_*O*_ mean observed heterozygosity, *H*_*E*_ mean expected heterozygosity, *A*_*n*_ mean frequency of null alleles, *SH* Shanghai, *HNHK* Haikou, Hainan, *ZJHZ* Hangzhou, Zhejiang, *SZSK* Shenzhen, Guangdong, *GDST* Shantou, Guangdong, *YNJH* Jinghong, Yunnan, *SXYC* Yuncheng, Shanxi, *SXYQ* Yangquan, Shanxi, *BJFT* Fengtai, Beijing, *SDJN* Jining, Shandong, *SXXA* Xian, Shaanxi, *GDGZ* Guangzhou, Guangdong, *HNSS* Sansha, Hainan, *GXNN* Nanning, Guangxi, *HNSY* Sanya, Hainan, *STWS* Waisha, Shantou, Guangdong, *GDZJ* Zhanjiang, Guangdong, information on the 17 field populations is presented in Additional file 1: Table S1

### Population genetic structure

In the analysis of population genetic structure, GDGZ was excluded due to the low number of individuals (*n* = 10). Therefore, a total of 443 individuals in 16 populations were analyzed for population genetic diversity. Pairwise comparisons indicate *F*_ST_ values were significantly different from zero, with the lowest value (0.012) found between SH with JZHZ, while the highest value (0.167) was between GDST with STWS (Table 2). We found no correlation between genetic differentiation (*F*_ST_) and geographical distance, which was confirmed by an R^2^ value of 0.029 (*P* = 0.785). All* F*_ST_ values within the population were greater than 0.1, except for that of GDST (0.087), with the range level from 0.271 (HNSS) to 0.582 (SH) (Table 2).

**Table 2:** Matrix of geographic distances (km) (above diagonal) and *F*_ST_ values (below diagonal and on the diagonal) among and within wild *Aedes albopictu*s populations collected across China

	SH	HNHK	ZJHZ	SZSK	GDST	YNJH	SXYC	SXYQ	BJFT	SDJN	SXXA	HNSS	GXNN	HNSY	STWS	GDZJ
SH	0.582	1696.33	178.66	1244.74	1014.42	2301.69	1055.44	1019.72	1051.93	637.83	1217.08	1868.70	1617.25	1896.61	1001.53	1578.29
HNHK	0.025	0.504	1517.69	472.25	765.37	1001.53	1669.86	2010.12	2281.17	1821.39	1596.63	423.98	362.12	211.12	778.73	134.63
ZJHZ	0.012	0.032	0.516	1066.80	842.7	2140.23	1012.96	1044.92	1128.61	667.95	1153.47	1694.27	1443.83	1718.01	830.10	1399.92
SZSK	0.046	0.071	0.038	0.479	299.62	1315.84	1423.41	1710.91	1946.14	1460.97	1405.35	651.82	574.60	657.86	312.28	383.32
GDST	0.078	0.054	0.089	0.141	0.087	1637.00	1409.93	1640.94	1837.16	1341.43	1435.27	856.67	857.40	936.97	13.80	682.88
YNJH	0.040	0.049	0.031	0.024	0.100	0.465	1754.62	2144.69	2465.50	2136.44	1584.13	1343.71	779.68	1003.33	1647.56	1004.30
SXYC	0.047	0.075	0.039	0.044	0.120	0.036	0.456	390.25	711.20	508.04	203.91	2030.34	1386.51	1872.34	1405.13	1537.15
SXYQ	0.080	0.091	0.072	0.032	0.164	0.043	0.082	0.371	323.02	382.34	571.66	2344.53	1750.58	2217.48	1633.54	1875.72
BJFT	0.052	0.062	0.042	0.020	0.126	0.026	0.053	0.041	0.295	496.08	896.46	2592.13	2042.51	2491.02	1828.10	2146.60
SDJN	0.057	0.080	0.050	0.016	0.155	0.032	0.048	0.034	0.030	0.321	707.05	2110.88	1615.96	2032.47	1332.26	1687.81
SXXA	0.054	0.075	0.045	0.014	0.149	0.030	0.048	0.046	0.030	0.015	0.299	1979.22	1287.14	1791.27	1432.37	1467.70
HNSS	0.066	0.059	0.064	0.042	0.129	0.040	0.081	0.061	0.045	0.047	0.051	0.271	786.08	340.44	870.00	528.37
GXNN	0.021	0.034	0.020	0.065	0.081	0.050	0.050	0.106	0.065	0.077	0.071	0.073	0.386	519.50	868.07	280.88
HNSY	0.105	0.103	0.102	0.070	0.160	0.073	0.100	0.059	0.077	0.068	0.075	0.075	0.129	0.305	950.69	344.92
STWS	0.072	0.087	0.061	0.023	0.167	0.047	0.061	0.045	0.041	0.028	0.035	0.067	0.090	0.083	0.327	659.59
GDZJ	0.063	0.074	0.052	0.018	0.149	0.031	0.057	0.031	0.031	0.014	0.020	0.045	0.081	0.054	0.018	0.401

*SH* Shanghai, *HNHK* Haikou, Hainan, *ZJHZ* Hangzhou, Zhejiang, *SZSK* Shenzhen, Guangdong, *GDST* Shantou, Guangdong, *YNJH* Jinghong, Yunnan, *SXYC* Yuncheng, Shanxi, *SXYQ* Yangquan, Shanxi, *BJFT* Fengtai, Beijing, *SDJN* Jining, Shandong, *SXXA* Xian, Shaanxi, *GDGZ* Guangzhou, Guangdong, *HNSS* Sansha, Hainan, *GXNN* Nanning, Guangxi, *HNSY* Sanya, Hainan, *STWS* Waisha, Shantou, Guangdong, *GDZJ* Zhanjiang, Guangdong, Information on the 17 field populations is presented in Additional file 1: Table S1

Results of Bayesian analysis implemented in STRUCTURE show that the proportion of individuals sharing coancestry in the 16 populations was not consistent with their geographic proximity. The natural logarithm of the likelihood of the data, i.e., ln (P(X/K)), increased from *K* = 1 to *K* = 3 and then gradually reached a plateau toward 16, suggesting that the genetic clustering of the 16 populations was 3 (Additional file 1: Figure S1). Individual mosquitoes from the 16 populations were then assigned to each of the 3 clusters with a certain probability value (Additional file 1: Figure S1, Table S4), and the pairwise and within cluster genetic differences (*F*_ST_) were calculated. The *F*_ST_ values between cluster I and clusters II and III were 0.110 and 0.114, respectively, while that between clusters II and III was 0.027. The *F*_ST_ values within the three clusters were 0.124, 0.048, and 0.080, respectively.

The coancestry of the populations in southern China were heterogeneous: i.e., populations in Hainan were tightly related to cluster II (green) (HNSY 95.8%, HNSS 76.6%) and cluster III (blue) (HNHK 57.7%), while populations in Guangdong were mainly related to cluster III (blue) (GDST 0.928) and cluster I (red) (STWS 79.7%, GDZJ 71.9%, SZSK 58.5%). Populations in the eastern part of China were weakly related to cluster III (SH 49.5%, JZHZ 48.1%), and populations in the northern part of China were mainly related to cluster I (BJFT 60.4%, SXXA 58.4%, SDJN 56.1%, SXYC 46.8%) (Additional file 1: Figure S2; Table S4).

### Effective population size

Estimates of longterm *Ne* varied considerably, depending on the method of calculation used. Using the heterozygote excess method resulted in all *Ne* estimates of infinity. Meanwhile, the linkage disequilibrium method, which used the lowest allele frequency of 0.01, yielded diverse *Ne* values across populations (Table 3). The effective sizes of the GDZJ and SH populations were infinite. However, the *Ne* value of the GDST population was 2.3 and this population’s wild-type frequency of F1534 was 100%.

**Table 3:** Estimated *Ne* across *Aedes albopictu*s populations based on the linkage disequilibrium method

	Population	*Ne*	95% *CI*
I (red)	STWS	38.3	20.3–121.9
	GDZJ	Infinite	179.5–Infinite
	BJFT	20.6	13.1–36.6
	SZSK	18.2	11.2–34.0
	SXXA	49.3	25.1–221.9
	SDJN	54.5	27.4–270.5
	SXYC	36.5	9.5–Infinite
II (green)	HNSY	33.5	19.6–75.3
	HNSS	353.5	18.6–Infinite
	SXYQ	11.0	2.9–410.3
	YNJH	233.8	54.2–Infinite
III (blue)	GDST	2.3	1.9–2.9
	GXNN	176.5	50.0–Infinite
	HNHK	127.9	39.8–Infinite
	SH	Infinite	4738.2–Infinite
	ZJHZ	59.5	28.7–443.5
	GDGZ	76.7	12.6–Infinite

*CI* confidence interval, *SH* Shanghai, *HNHK* Haikou, Hainan, *ZJHZ* Hangzhou, Zhejiang, *SZSK* Shenzhen, Guangdong, *GDST* Shantou, Guangdong, *YNJH* Jinghong, Yunnan, *SXYC* Yuncheng, Shanxi, *SXYQ* Yangquan, Shanxi, *BJFT* Fengtai, Beijing, *SDJN* Jining, Shandong, *SXXA* Xian, Shaanxi, *GDGZ* Guangzhou, Guangdong, *HNSS* Sansha, Hainan, *GXNN* Nanning, Guangxi, *HNSY* Sanya, Hainan, *STWS* Waisha, Shantou, Guangdong, *GDZJ* Zhanjiang, Guangdong, information on the 17 field populations is presented in Additional file 1: Table S1

### Correlation between the intrapopulation variation and knockdown resistance mutations

Pearson correlation analysis of *F*_ST_ and the frequency of F1534 wild-type within 14 *Ae. albopictus* populations yielded a correlation coefficient of − 0.39 (*P* = 0.17), indicating no significant correlation. However, we did observe the following trend: the greater the genetic difference (*F*_ST_), the lower the wild-type frequency of F1534 (Additional file 1: Figure S3). In the GDST population, codon 1534 in *VGSC* was entirely wild-type (100%), and the population had the lowest *F*_ST_ (0.087). Meanwhile, the ZJHZ population had the highest (91.55%) F1534 mutant allele frequency, and its *F*_ST_ value was also large (0.516) (Table 2).

## Discussion

*Ae. albopictus* has long been considered of secondary importance as a vector for dengue transmission. However, this species is the most important vector in China because it is widespread, while the distribution of the typical primary vector, *Ae. aegypti*, is limited. Moreover, recent dengue outbreaks in China have been caused by *Ae. albopictus* [2]. Therefore, more knowledge on the genetic diversity and genetic structure of this mosquito species will help researchers evaluate the risk of disease transmission and insecticide resistance spread, as well as identify the origins and frequencies of introductions.

In this study, we analyzed the population genetics of *Ae. albopictus* collected from the different climate regions in China based on the 8 polymorphic microsatellite loci. Most (91%) of the genetic variation occurred within individuals, whereas only about 9% of the total variation was detected among populations. We found no relationship between genetic distance and geographical distance, and populations in the southern part of China tended to be more heterogeneous. Within a population, we observed that the greater the genetic difference (*F*_ST_), the lower the wild-type frequency of F1534 of *VSGC*.

Sampling strategy and geographic coverage greatly influence the analysis and interpretation of the data generated from the samples. In this study, *Ae. albopictus* mosquitoes were collected from 17 sites in 11 provinces (municipalities), encompassing the main distribution range of *Ae. albopictus* in China (i.e., from 16°N to 40°N [4]), which includes tropical, subtropical, and temperate climate zones. In addition, it is crucial to clearly distinguish between the type of collected samples (i.e., eggs, larvae, or flying adults), because *Ae. albopictus* larvae or eggs collected from the same breeding site are likely to belong to the same progeny. We avoided this potential drawback by collecting a few individuals per breeding site and setting multiple traps throughout the sampling site.

The following are key features of useful genetic markers: selective neutrality, ease of scoring in all specimens of the species, and sufficient variability to allow for measuring genetic differentiation and genetic clustering of individuals. Previous studies have reported on more than 10 microsatellite DNA loci for *Ae. albopictus* populations, and we found eight of them were highly polymorphic and amplified efficiently, and these were used for exploring the population genetic structure of *Ae. albopictus* in China. Although their chromosomal locations are unknown, we observed no consistent pattern of linkage disequilibrium between any particular pair of loci, suggesting that they are at least statistically independent and are likely distributed genome-wide. However, in the GDGZ population, we were unable to amplify some microsatellite loci in many individuals, resulting in invalid data. The high amplification failure rate in GDGZ was unanticipated and may be due to inherent genetic differences in *Ae. albopictus* samples, which have been suggested in other studies [53] and are thought to influence dengue virus susceptibility [54].

We observed high allelic diversity and heterozygosity in most of the populations (*H*_O_ = 0.32–0.57), which is similar to the levels of diversity in the *Ae. albopictus* population at the coastal areas in southern China (*H*_O_ = 0.384–0.641) [25], in 17 populations from 3 climatic regions of China (*H*_O_ = 0.467–0.627) [9], in populations from 34 localities across China (*H*_O_ = 0.551–0.633) [29], and in eight samples from Thailand, Réunion, and Northern Italy (*H*_O_ = 0.22–0.28). In China, *Ae. albopictus* occurs in different climate zones, with populations undergoing marked seasonal variations in abundance, in which high densities are reached only during the summer months. The high levels of genetic diversity suggest that *Ae. albopictus* is able to maintain a relatively large effective population size, despite the seasonal changes in temperature.

Notably, we observed the minimum *F*_ST_ value (0.087) in the GDST population, which carried no mutation in F1534 [8]. As this population is located close to seaside wharf, we speculate that the GDST population consists of mosquitoes that originated from ships and that colonized the local environment, which was suitable for the survival of *Ae. albopictus*. This population became abundant and because the time of colonization time may be recent or it may be derived from a single invasion, the genetic difference within the population is small, with 92.8% of the individuals belonging to cluster III. These attributes are consistent with the founder effect. Oral interviews revealed that almost no insecticides have been used in the GDST area, thus the alleles of F1534 are all wild-type. Similarly, HNSS was collected from a tropical island far from the Chinese mainland and with no source of fresh water. This population is likely to have been transported to the island by ships from Hainan Island, thus both the HNSS and HNSY populations belong to cluster II.

The results of STRUCTURE analysis show that these *Ae. albopictus* populations are divided into three clusters. Each cluster could be considered as a gene pool, the northern Chinese *Ae. albopictus* populations belong to gene pool I while the eastern populations belong to gene pool III. Meanwhile, the southern populations consist of three different gene pools. The genetic differences among the three gene pools are small. The FST values between cluster I and clusters II and III were 0.110 and 0.114, respectively, while that between clusters II and III was 0.027.

*Ae. albopictus* is also known as the Asian tiger mosquito, and it has been described as one of the 100 worst invasive species in the world. The species originated from South and East Asia, and it has spread throughout the world mostly since the second half of the twentieth century. It is now found on every continent except Antarctica [55]. Areas that have been colonized by *Ae. albopictus* include disparate environments such as tropical South America, Africa, and the most temperate areas of Northern America and Europe. In China, the species likely invaded the south first, colonizing the tropical and subtropical regions. From these regions, *Ae. albopictus* can colonize new areas and expand its population. In addition, *Ae. albopictus* populations from other places may have been able to invade many times; therefore, the genetic pools of the population in the southwest and south are relatively complex, as shown by STRUCTURE analysis.

Analysis of the likely migration route of *Ae. albopictus* in China indicates that the southern population of *Ae. albopictus* was introduced into the eastern and northern (the temperate regions) regions through the movement of people and goods. This describes a chaotic propagule distribution mediated by human activity, and it resulted in a relatively simple gene pool. Thus, *Ae. albopictus* populations in eastern and northern China should be temperate-diapausing, and there was sufficient gene flow with tropical nondiapausing populations. In particular, the photoperiodical diapause, which has a demonstrated genetic basis, seems to be an important component of climatic adaptation that favors the invasive success of *Ae. albopictus*. Conversely, anthropogenic activities also create new breeding and trophic niches of adaptation in close proximity to human living sites, which impacts the relationship between mosquitoes and humans.

Pearson correlation analysis found no correlation between *kdr* mutation and genetic structure. However, we did observe that low *F*_ST_ is associated with more wild-type, while high *F*_ST_ is associated with less wild-type. The mechanism of *kdr* mutation, which is closely related to insecticide resistance, is a very complex issue. This intrinsic interaction may be influenced by other important influencing factors, including climate variations, geographic distance, and the properties of insecticides used for mosquito control in different regions. Therefore, understanding the relationship between genetic structure and *kdr* mutations requires careful analysis, with consideration of case-specific factors. To clarify this issue, more samples need to be collected over a wider geographical area, ideally over multiple years, along with insecticide usage history. Perhaps in the future, more definite conclusions about the mechanism of *kdr* evolution in *Ae. albopictus* populations with different genetic backgrounds can be compared using a laboratory model under artificial insecticide pressure.

The main limitation of this study is the paucity of samples considering the large geographic area of China. Thus, the sampling areas should be expanded to obtain more detailed data. Therefore, we will continue to collect large numbers of samples of *Ae. albopictus* in the future to obtain more population data that can be used to further explore the correlation between resistance mutations and genetic background.

## Conclusions

We observed a very low degree of genetic divergence among *Ae. albopictus* populations in China, and we found no relationship between genetic and geographical distance. These populations consist of three gene pools. The gene pools of the northern and eastern Chinese populations were relatively homogeneous, while the southern populations were heterogeneous. Greater genetic difference within a population tended to be associated with a higher mutation rate of knockdown resistance, which deserves further attention and exploration.

## Supplementary Information

**Additional file 1: Figure S1.** Bayesian cluster analysis using STUCTURE. Graphical representation of the data set for the most likely K, where each color corresponds to a suggested cluster and each individual is represented by a vertical bar. The X-axis corresponds to the population codes, while the Y-axis represents the probability of assignment of an individual to each cluster. **Figure S2.** The collection sites for *Aedes albopictus* in China. The composition of the three clusters in each site is represented by a pie chart, with red, green, and blue representing clusters I, II, and III, respectively. The populations in the dotted circle belong to different clusters. **Figure S3.** Scatter plot of the Pearson correlation between F1534 wild-type frequency and FST value within 14 *Ae. albopictus* populations. **Table S1.** Sampling information of *Aedes albopictus* populations in China. **Table S2.** Primer sequences of 8 microsatellite loci of *Aedes albopictus*. **Table S3.** Number of alleles per population at each microsatellite locus. **Table S4.** Average coefficient of ancestry obtained from a STRUCTURE run with K = 3 for 443 individuals of *Aedes albopictus* from 16 samples collected in China.

## Data Availability

Data supporting the conclusions are included within the article and additional files. The datasets used and/or analyzed during this study are available from the corresponding author upon reasonable request.

## Abbreviations

*kdr*: Knockdown resistance
*VGSC*: Voltage-gated sodium channels
*F*_ST_: Fixation index
COI: Cytochrome c oxidase subunit I
