# Supplementary material for: Genetic structure of Aedes albopictus (Diptera: Culicidae) populations in China and relationship with the knockdown resistance mutations
Source: Infect Dis Poverty. 2023 May 5;12:46. doi: 10.1186/s40249-023-01096-x (PMC10161448; doi:10.1186/s40249-023-01096-x)
Supplement: Supplementary file 1 — Additional file 1: Figure S1. Bayesian cluster analysis using STUCTURE. Graphical representation of the data set for the most likely K, where each color corresponds to a suggested cluster and each individual is represented by a vertical bar. The X-axis corresponds to the population codes, while the Y-axis represents the probability of assignment of an individual to each cluster. Figure S2. The collection sites for Aedes albopictus in China. The composition of the three clusters in each site is represented by a pie chart, with red, green, and blue representing clusters I, II, and III, respectively. The populations in the dotted circle belong to different clusters. Figure S3. Scatter plot of the Pearson correlation between F1534 wild-type frequency and FST value within 14 Ae. albopictus populations. Table S1. Sampling information of Aedes albopictus populations in China. Table S2. Primer sequences of 8 microsatellite loci of Aedes albopictus. Table S3. Number of alleles per population at each microsatellite locus. Table S4. Average coefficient of ancestry obtained from a STRUCTURE run with K = 3 for 443 individuals of Aedes albopictus from 16 samples collected in China. [file 40249_2023_1096_MOESM1_ESM.docx]

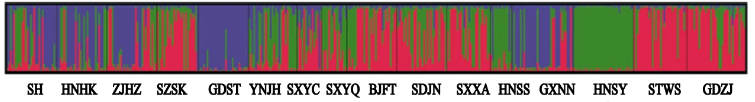


**Figure S1 Bayesian cluster analysis using STUCTURE.** Graphical representation of the data set for the most likely *K* (*K* = 3), where each color corresponds to a suggested cluster and each individual is represented by a vertical bar. The X-axis corresponds to the population codes, while the Y-axis represents the probability of assignment of an individual to each cluster.

**Figure S2. The collection sites for *Aedes albopictus* in China.** The composition of the three clusters in each site is represented by a pie chart, with red, green, and blue representing clusters I, Ⅱ, and Ⅲ, respectively (for details see Table 1, Table S4). The populations in the dotted circle belong to different clusters.


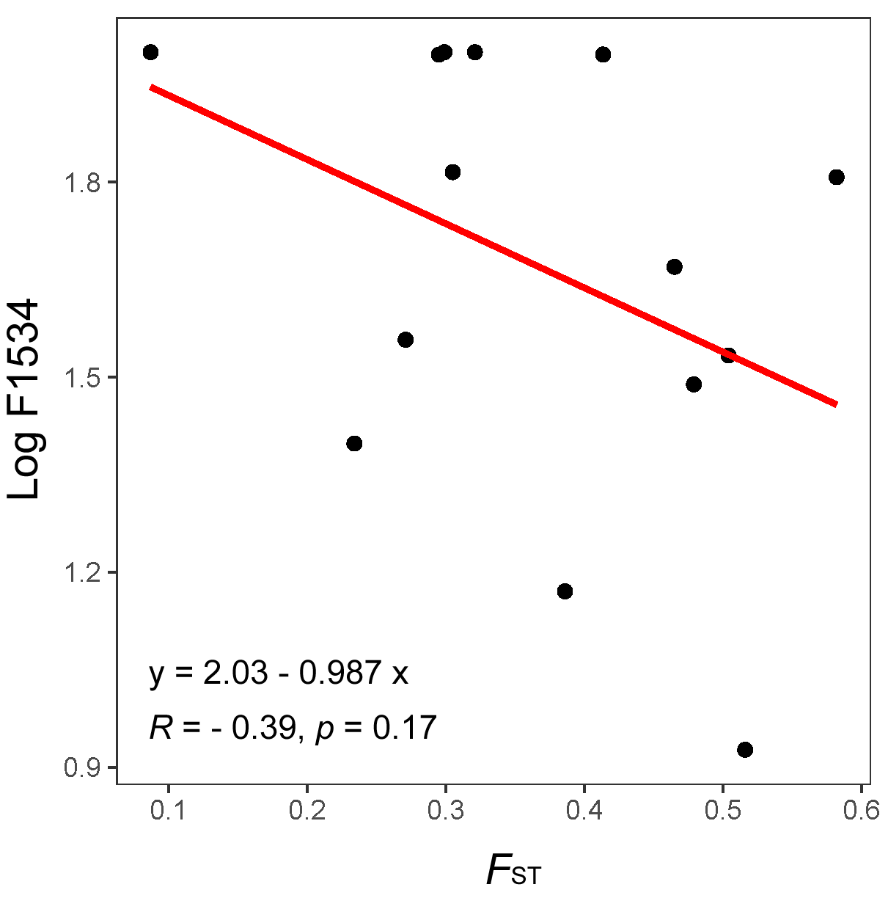


**Figure S3. Scatter plot of the Pearson correlation between F1534 wild-type frequency and *F*_ST_ value within 14 *Ae. albopictus* populations (R =** **−0.39, *P* = 0.17).**

**Table S1. Sampling information of *Aedes albopictus* populations in China.**

| Collection site | Population codes | Sample size | | Latitude | Longitude | Date of collection |
| --- | --- | --- | --- | --- | --- | --- |
| Baoshan, Shanghai | SH | | 30 | 31°24′ N | 121°29′ E | August 2017 |
| Haikou, Hainan | HNHK | | 30 | 20°02′ N | 110°11′ E | June–October 2017 |
| Hangzhou, Zhejiang | ZJHZ | | 30 | 30°14′ N | 120°12′ E | September 2017 |
| Shekou, Shenzhen, Guangdong | SZSK | | 25 | 22°29′ N | 113°54′ E | June 2018 |
| Shantou, Guangdong | GDST | | 30 | 23°21′ N | 116°40′ E | May 2018 |
| Jinghong, Yunnan | YNJH | | 29 | 22°02′ N | 100°47′ E | October 2016; September 2017 |
| Yuncheng, Shanxi | SXYC | | 15 | 35°01′ N | 111°0′ E | June–October 2017 |
| Yangquan, Shanxi | SXYQ | | 15 | 37°51′ N | 113°34′ E | June–October 2017 |
| Fengtai, Beijing | BJFT | | 30 | 39°51′ N | 116°16′ E | July 2018 |
| Jining, Shandong | SDJN | | 30 | 35°24′ N | 116°34′ E | July 2018 |
| Xian, Shaanxi | SXXA | | 26 | 34°20′ N | 108°56′ E | September–October 2017; June–October 2018 |
| Guangzhou, Guangdong | GDGZ | | 10 | 23°07′ N | 113°15′ E | November 2019 |
| Sansha, Hainan | HNSS | | 13 | 16°49′ N | 112°20′ E | April 2019 |
| Nanning, Guangxi | GXNN | | 37 | 22°47′ N | 108°19′ E | May 2020 |
| Sanya, Hainan | HNSY | | 36 | 18°15′ N | 109°30′ E | June 2019 |
| Waisha, Shantou, Guangdong | STWS | | 32 | 23°26′ N | 116°46′ E | March 2021 |
| Zhanjiang, Guangdong | GDZJ | | 35 | 21°13′ N | 110°27′ E | March 2021 |

**Table S2. Primer sequences of 8 microsatellite loci of *Aedes albopictus*.**

| Locus | Primer (5’→3’) | Ta (℃) | GenBank accession number |
| --- | --- | --- | --- |
| Alb-di-4 | F：TGGCGACCTATTATACCCGC | 50 | KF146971 |
|  | R：CAACTCGTTCCTTGACCGTG |  |  |
| Alb-di-6 | F：TCTTCATCTACGCTGTGCTC | 50 | KF146972 |
|  | R：GACGCCAATCCGACAAAGTC |  |  |
| Alb-tri-6 | F：AGCACGAGTACAGAATGTGC | 50 | KF146974 |
|  | R：TGGCCTCCTACCGTTTATCTG |  |  |
| Alb-tri-41 | F：GATCGATTTGGGAGCTTCTG | 50 | KF146983 |
|  | R：GAACCTCTTCTCGCTTGGCT |  |  |
| Alb-tri-46 | F：TTCACAACATACGGAATCGC | 50 | KF146975 |
|  | R：GGTCCGGTGTAATAGCCTCC |  |  |
| AealbA9 | F：TGGGACAAGAGCTGAAGGAT | 52 | DQ366022 |
|  | R：CTCGTTCTCTACTCTCTCCGTT |  |  |
| AealbB52 | F：GGGTCTAGAAGTAATAGCGATCG | 50 | DQ366024 |
|  | R：GCATTCTTTGCTTCTGTTTGC |  |  |
| AealbF3 | F：CTCGTGAGTACGTTCCGTGA | 53 | DQ366027 |
|  | R：AGGGAAACAAGGACTTCATCA |  |  |

**Table S3. Number of alleles per population at each microsatellite locus.**

| Populations | Alb-di-4 | Alb-di-6 | Alb-tri-6 | Alb-tri-41 | Alb-tri-46 | AealbA9 | AealbB52 | AealbF3 |
| --- | --- | --- | --- | --- | --- | --- | --- | --- |
| SH | 5 | 7 | 10 | 6 | 6 | 11 | 3 | 3 |
| HNHK | 3 | 8 | 9 | 7 | 7 | 11 | 3 | 4 |
| ZJHZ | 2 | 9 | 11 | 5 | 8 | 10 | 3 | 3 |
| SZSK | 2 | 8 | 10 | 5 | 7 | 8 | 3 | 4 |
| GDST | 4 | 6 | 5 | 7 | 5 | 5 | 3 | 4 |
| YNJH | 4 | 10 | 9 | 7 | 8 | 10 | 4 | 6 |
| SXYC | 3 | 5 | 8 | 6 | 5 | 7 | 5 | 3 |
| SXYQ | 4 | 4 | 10 | 4 | 6 | 6 | 1 | 2 |
| BJFT | 4 | 5 | 8 | 5 | 6 | 11 | 2 | 3 |
| SDJN | 3 | 8 | 10 | 7 | 8 | 8 | 3 | 3 |
| SXXA | 4 | 7 | 8 | 7 | 8 | 11 | 3 | 4 |
| GDGZ | 5 | 7 | 7 | 5 | 6 | 10 | 2 | 4 |
| HNSS | 5 | 6 | 6 | 4 | 5 | 10 | 3 | 2 |
| GXNN | 5 | 8 | 9 | 5 | 6 | 10 | 3 | 4 |
| HNSY | 4 | 7 | 11 | 6 | 8 | 3 | 1 | 6 |
| STWS | 2 | 6 | 9 | 5 | 6 | 9 | 1 | 6 |
| GDZJ | 3 | 8 | 12 | 8 | 9 | 13 | 2 | 4 |
| Total | 8 | 15 | 18 | 10 | 15 | 27 | 7 | 11 |

**Table S4. Average coefficient of ancestry obtained from a STRUCTURE run with *K* = 3 for 443 individuals of *Aedes albopictus* from 16 samples collected in China.**

| Cluster | Population | I (red) | Ⅱ (green) | Ⅲ (blue) |
| --- | --- | --- | --- | --- |
| I (red) | STWS | 79.7% | 17.1% | 3.2% |
|  | GDZJ | 71.9% | 25.3% | 2.8% |
|  | BJFT | 60.4% | 35.5% | 4.0% |
|  | SZSK | 58.5% | 34.0% | 7.5% |
|  | SXXA | 58.4% | 36.8% | 4.9% |
|  | SDJN | 56.1% | 39.4% | 4.4% |
|  | SXYC | 46.8% | 27.9% | 25.3% |
| Ⅱ (green) | HNSY | 2.2% | 95.8% | 2.0% |
|  | HNSS | 8.6% | 76.6% | 14.8% |
|  | SXYQ | 31.6% | 59.6% | 8.8% |
|  | YNJH | 22.8% | 54.1% | 23.1% |
| Ⅲ (blue) | GDST | 2.6% | 4.6% | 92.8% |
|  | GXNN | 17.3% | 17.1% | 65.5% |
|  | HNHK | 14.4% | 27.9% | 57.7% |
|  | SH | 24.1% | 26.3% | 49.5% |
|  | ZJHZ | 28.4% | 23.6% | 48.1% |
